# Supplementary material for: The stability of m6A-marked transcripts is linked to cell identity in planarians
Source: Nucleic Acids Res. 2026 Sep 22;54(18):gkag892. doi: 10.1093/nar/gkag892 (PMC13594981; doi:10.1093/nar/gkag892)
Supplement: Supplementary file 1 [file gkag892_supplemental_files.zip › Supplementary_Material_revised_Aug6_final.pdf]

Supplementary Material

The stability of m^6A-marked transcripts is linked to cell identity in planarians

Constantin Höhn[1,†], Andreas Pittroff[1,†], Anne-Sophie Gribling-Burrer[2], Lisa Hülsmann[3], Redmond P. Smyth[2] and Claus-D. Kuhn[1,*]

[1] RNA Biochemistry, Medical Campus Upper Franconia (MCO), University of Bayreuth, Bayreuth, Germany

[2] Genome Biology of Viruses, Architecture et Réactivitié de l'ARN, Université de Strasbourg, CNRS, Institute of Molecular and Cellular Biology (IBMC), Strasbourg, France

[3] Ecosystem Analysis and Simulation, Bayreuth Center of Ecology and Environmental Research (BayCEER), University of Bayreuth, Germany

† These authors contributed equally to this work.

* To whom correspondence should be addressed: claus.kuhn@uni-bayreuth.de

<u>List of supplementary material:</u>

Supplementary Figures S1 – S6

Supplementary Tables S1 – S7

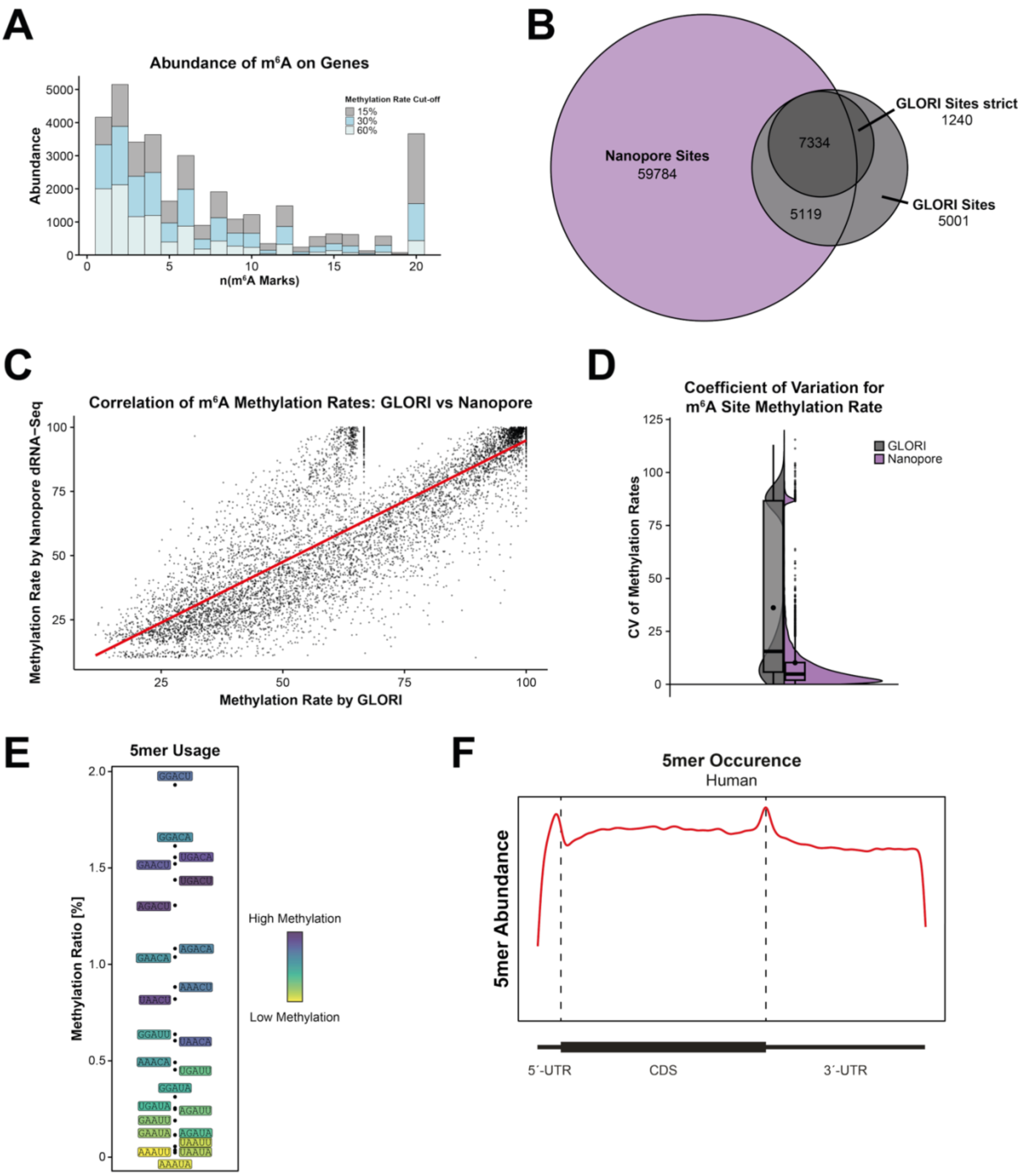

A
Abundance of m⁶A on Genes
Methylation Rate Cut-off
15%
30%
60%
Abundance
n(m⁶A Marks)
B
Nanopore Sites
59784
7334
5119
GLORI Sites strict
1240
GLORI Sites
5001
C
Correlation of m⁶A Methylation Rates: GLORI vs Nanopore
Methylation Rate by Nanopore dRNA−Seq
Methylation Rate by GLORI
D
Coefficient of Variation for
m⁶A Site Methylation Rate
GLORI
Nanopore
CV of Methylation Rates
E
5mer Usage
Methylation Ratio [%]
High Methylation
Low Methylation
GGACU
GGACA
UGACA
GAACU
UGACU
AGACU
AGACA
GAACA
AAACU
UAACU
GGAUU
UAACA
AAACA
UGAUU
GGAUA
UGAUA
AGAUU
GAAUU
GAAUA
AGAUA
UAAUU
AAAUU
UAAUA
AAAUA
F
5mer Occurence
Human
5mer Abundance
5´-UTR
CDS
3´-UTR

Figure S1. m^{6}A site occurrence in planarians. (A) Number of m^{6}A sites on planarian genes at different methylation rates. Median number of sites per gene was 7 at 15%, 4 at 30%, and 4 at 60% methylation rate, respectively. **(B)** Comparison of planarian m^{6}A sites detected by Nanopore dRNA-Seq (lilac) and GLORI (grey as reported in Yesharim et al. 2026, dark grey with a filter for coverage in at least two of three replicates (strict)). 70.9% of GLORI sites and 88.0% of strict GLORI sites were captured by Nanopore dRNA-Seq. **(C)** Correlation of mean methylation rates for m^{6}A sites detected simultaneously in Nanopore dRNA-Seq and GLORI. The scatter plot displays mean methylation rates from Nanopore dRNA-Seq on the y-axis and GLORI on the x-axis, with each dot representing an individual m^{6}A site detected by both methods. Mean methylation rates had a strong positive correlation (red line; $r = 0.825$; 95% CI 0.818–0.833; $p < 2.2 \times 10^{-16}$). A distinct cluster of points forms above the main diagonal distribution, truncated at 66.6%, corresponding to cases where one of the three GLORI replicates reported zero methylation (e.g., two replicates at 100% and one at 0%). **(D)** Coefficient of variation (CV) of respective methylation rates was calculated for all m^{6}A sites detected by Nanopore dRNA-Seq (purple) and GLORI (dark grey). Shared Sites displayed significantly lower CVs in Nanopore methylation rates than in GLORI methylation rates ($p < 2.2 \times 10^{-16}$, Wilcoxon rank-sum test). **(E)** Methylation ratio of individual 5-mers (defined as methylated 5-mer/occurrence of 5-mer) coincides with methylation rate of respective 5-mers, meaning that 5-mers with lower methylation rates are also methylated less frequently in total. Displayed colors are equivalent to Fig.1F. **(F)** Metagene plot of all 5-mers making up the human DRACH motif shows that motifs are distributed more evenly along human genes with less pronounced enrichment around the CDS-3'UTR border.

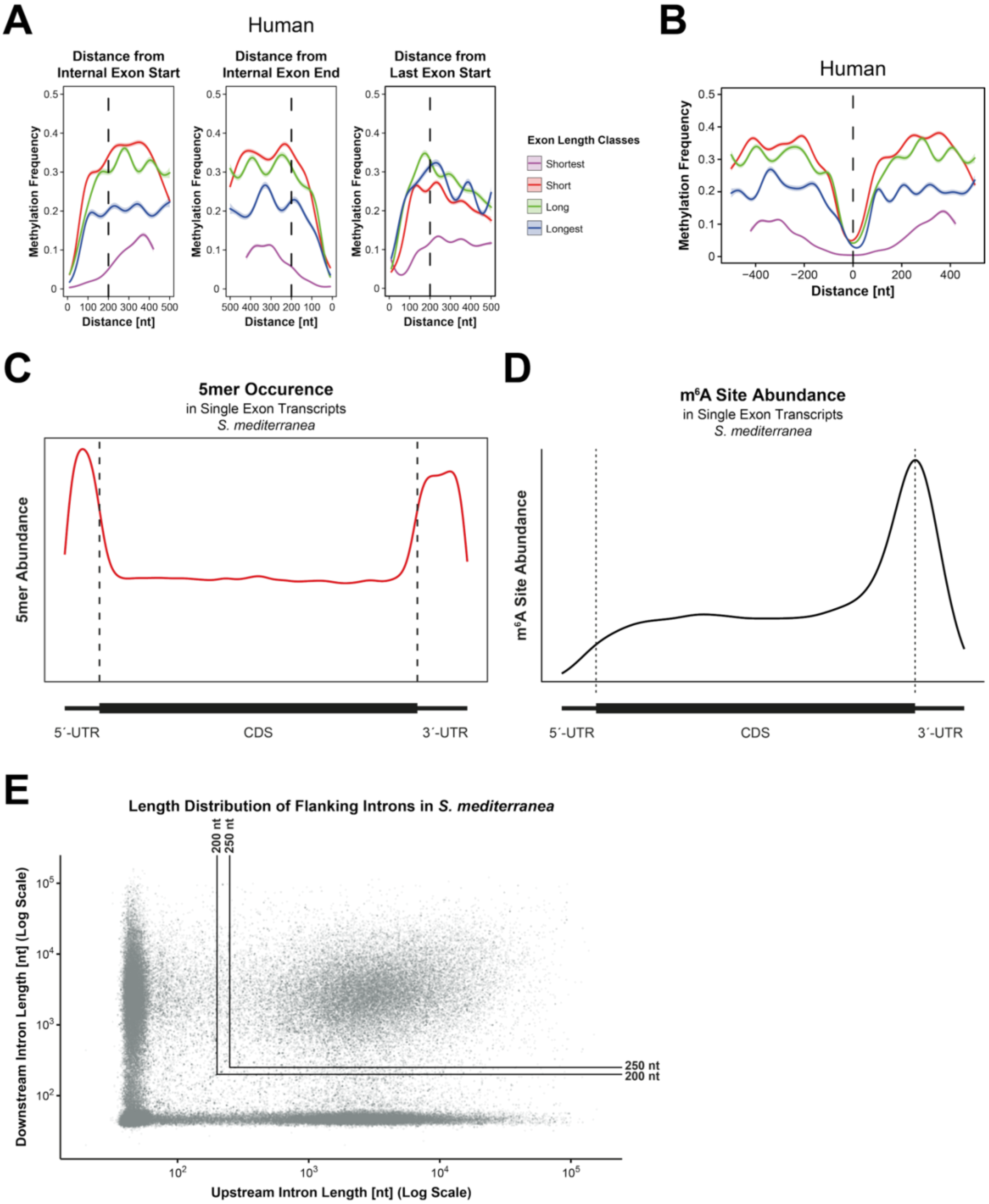

A
Human
Distance from Internal Exon Start
Distance from Internal Exon End
Distance from Last Exon Start
Methylation Frequency
Distance [nt]
Exon Length Classes
Shortest
Short
Long
Longest
B
Human
Methylation Frequency
Distance [nt]
C
5mer Occurence
in Single Exon Transcripts
S. mediterranea
5mer Abundance
5´-UTR
CDS
3´-UTR
D
m6A Site Abundance
in Single Exon Transcripts
S. mediterranea
m6A Site Abundance
5´-UTR
CDS
3´-UTR
E
Length Distribution of Flanking Introns in S. mediterranea
200 nt
250 nt
Downstream Intron Length [nt] (Log Scale)
Upstream Intron Length [nt] (Log Scale)

Figure S2. Methylation frequency in human data recapitulates the EJC exclusion model. (A) Generalized additive models (GAMs) of human methylation frequency towards exon boundaries reveal depletion of m^6A in a ~200 nt window towards internal exon starts and ends, and last exon starts, recapitulating the m^6A exclusion zone found in humans. GAMs were fitted for exon length classes defined by length quartiles. 95% confidence intervals of the model fit are depicted as shaded area; methylation frequency was tested in bins of 10 nt. **(B)** GAMs across human splice junctions recap the exclusion zone, which is centered on the splice junction and spans from ~ -200 nt to ~ +200 nt in humans. GAMs were fitted for exon length classes defined by length quartiles. 95% confidence intervals of the model fit are depicted as shaded area; methylation frequency was tested in bins of 10 nt. **(C-D)** Metagene plot of m^6A sites in planarian single exon transcripts does not reveal a distinguished distribution of motif occurrences (C), however m^6A site abundance in the CDS is elevated (D) compared to multi-exon transcripts (Fig. 1G, D). **(E)** Length distribution of exon-flanking introns in *Schmidtea mediterranea*. The scatter plot displays downstream intron length on the y-axis and upstream intron length on the x-axis, with each dot representing an individual internal exon of *Schmidtea mediterranea*. The x- and y-axes are shown in log scale. Black lines demarcate the transition zone between intron- and exon-defined splicing ranging from 200 to 250 nt. 78.5% of internal exons in *Schmidtea mediterranea* are flanked by at least one short intron.

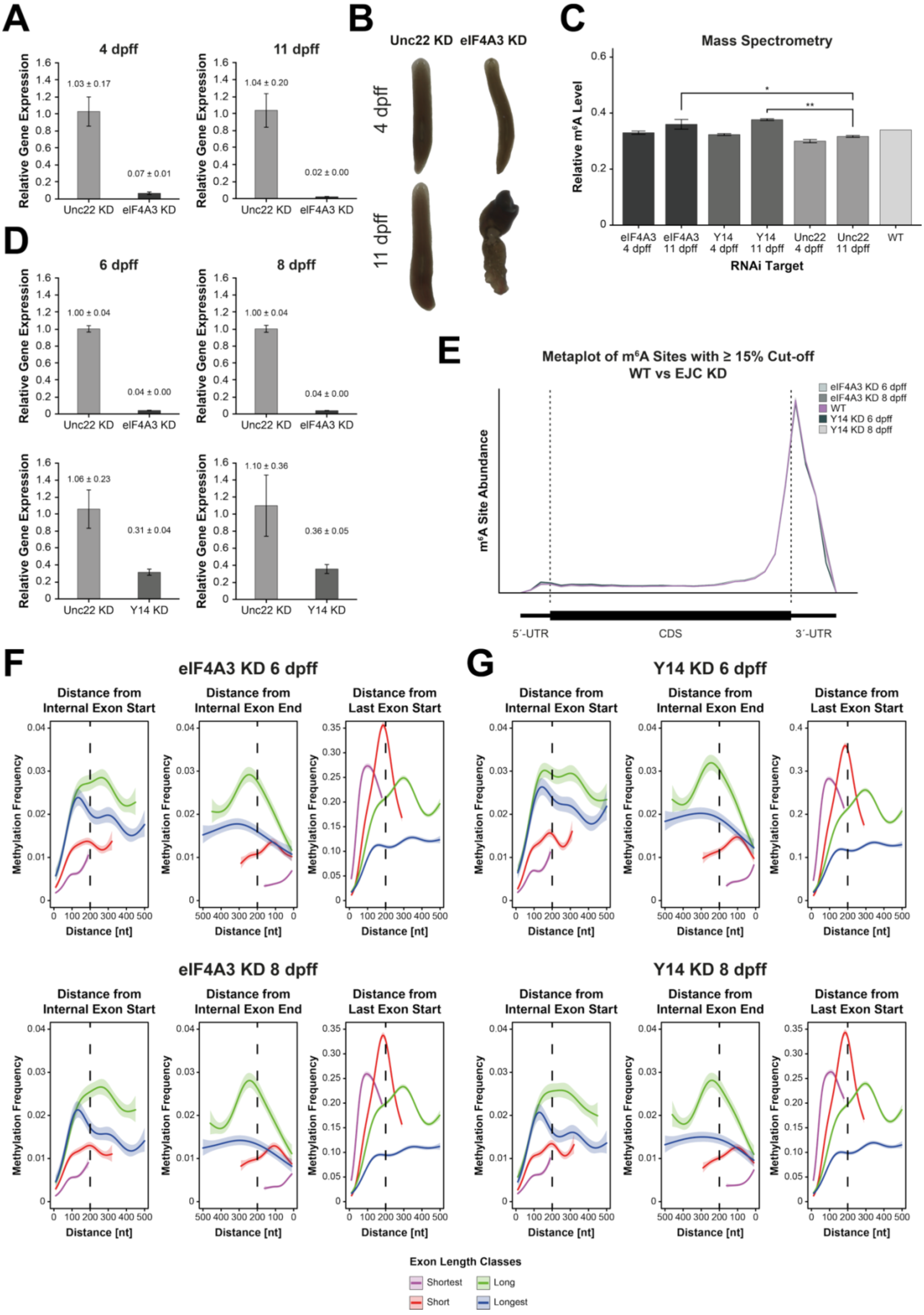
A
4 dpff
11 dpff
Relative Gene Expression
1.03 ± 0.17
0.07 ± 0.01
1.04 ± 0.20
0.02 ± 0.00
Unc22 KD
eIF4A3 KD
B
Unc22 KD
eIF4A3 KD
4 dpff
11 dpff
C
Mass Spectrometry
Relative m6A Level
eIF4A3 4 dpff
eIF4A3 11 dpff
Y14 4 dpff
Y14 11 dpff
Unc22 4 dpff
Unc22 11 dpff
WT
RNAi Target
D
6 dpff
8 dpff
1.00 ± 0.04
0.04 ± 0.00
1.06 ± 0.23
0.31 ± 0.04
1.10 ± 0.36
0.36 ± 0.05
Y14 KD
E
Metaplot of m6A Sites with ≥ 15% Cut-off
WT vs EJC KD
m6A Site Abundance
eIF4A3 KD 6 dpff
eIF4A3 KD 8 dpff
WT
Y14 KD 6 dpff
Y14 KD 8 dpff
5´-UTR
CDS
3´-UTR
F
eIF4A3 KD 6 dpff
eIF4A3 KD 8 dpff
G
Y14 KD 6 dpff
Y14 KD 8 dpff
Distance from Internal Exon Start
Distance from Internal Exon End
Distance from Last Exon Start
Methylation Frequency
Distance [nt]
Exon Length Classes
Shortest
Short
Long
Longest

Figure S3. Validation of EJC component knockdowns and impact on exclusion zone. (A) Validation of eIF4A3 knockdown in pre-experiments. Relative eIF4A3 mRNA levels were quantified by qPCR at 4 and 11 days post first feeding (dpff) following RNAi treatment. Efficient and sustained knockdown of eIF4A3 was observed compared to the Unc22 control. Y14 knockdown is not shown due to technical issues in the initial qPCR setup. Bars show mean ± SE. **(B)** Phenotypic consequences of eIF4A3 knockdown during planarian homeostasis. Representative images of animals subjected to RNAi against *unc22* or *eIF4A3* at 4 and 11 dpff. While control animals maintained normal morphology, *eIF4A3* knockdown resulted in pronounced homeostatic defects over time, supporting effective knockdown. Y14 knockdown animals are not shown. **(C)** Global m^6A levels were quantified by mass spectrometry following RNAi-mediated depletion of the EJC components eIF4A3 and Y14 at the indicated time points. Relative m^6A levels remained comparable to wild-type and Unc22 controls, indicating that EJC depletion does not lead to elevated global m^6A levels. Bars show mean ± SE. Significance was tested using ANOVA and Tukey HSD test; p-values were corrected for multiple testing by Studentized range distribution; adjusted p-value: ** ≤ 0.01; * ≤ 0.05. **(D)** qPCR validation of *eIF4A3* and *y14* knockdown in samples used for nanopore sequencing. Relative eIF4A3 and Y14 mRNA levels were quantified at 6 and 8 dpff following RNAi treatment. Robust knockdown of both targets was achieved in these samples, which were subsequently used for nanopore direct RNA sequencing. Bars show mean ± SE. **(E)** Metagene analysis does not identify differential m^6A site abundance profiles between wildtype and EJC depleted samples. **(F-G)** GAMs of methylation frequencies in *eIF4A3* (RNAi) and *y14* (RNAi) knockdowns towards exon boundaries at 6 dpff and 8 dpff did not reveal considerable differences in methylation frequencies compared to wildtype. GAMs were fitted for exon length classes defined by length quartiles in WT. 95% confidence intervals of the model fit are depicted as shaded area; methylation frequency was tested in bins of 10 nt. The number of exons and transcripts, on which this analysis is based on, is listed in Supplementary Table S5.

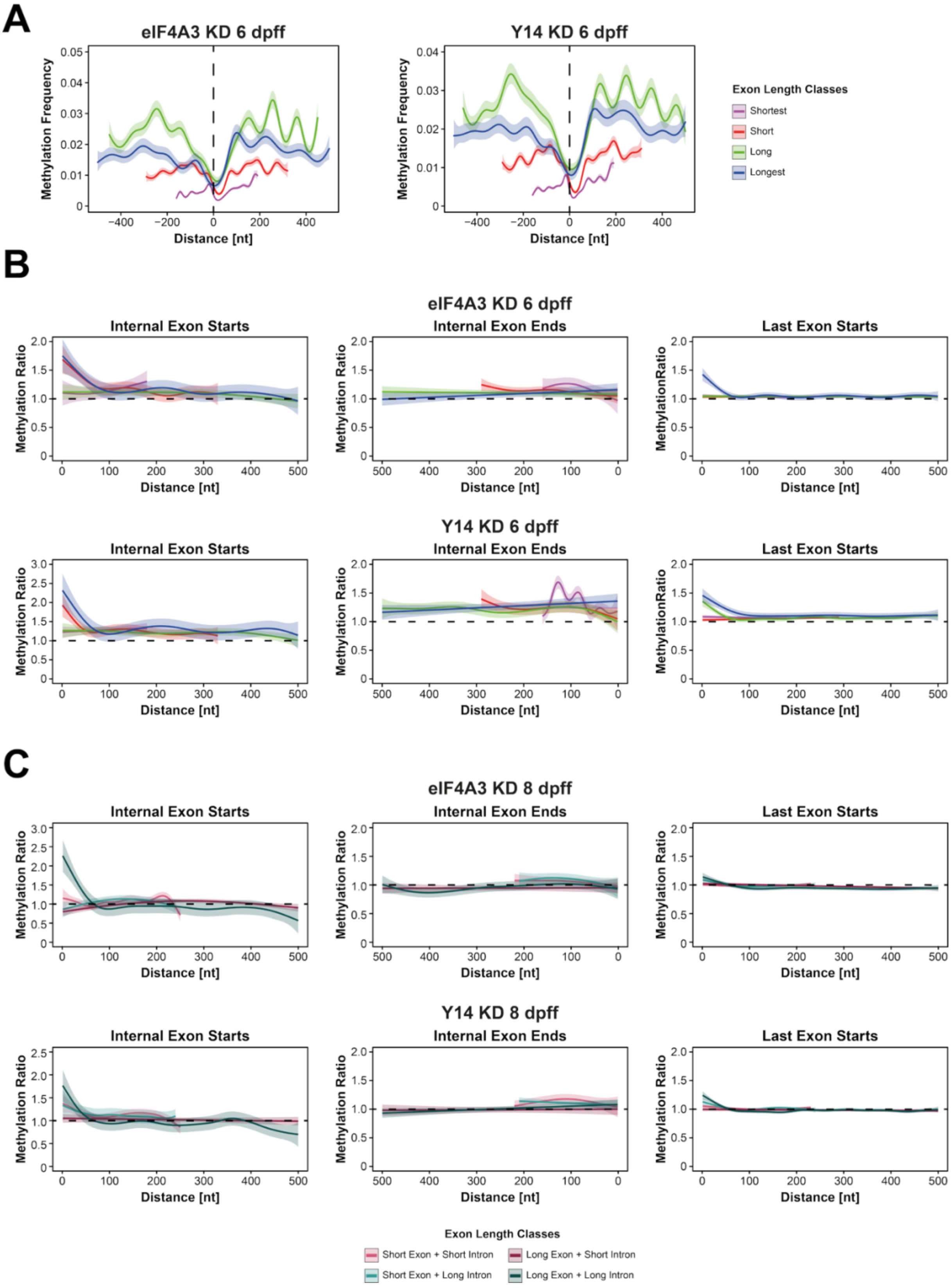
A
eIF4A3 KD 6 dpff
Y14 KD 6 dpff
Methylation Frequency
Distance [nt]
Exon Length Classes
Shortest
Short
Long
Longest
B
eIF4A3 KD 6 dpff
Y14 KD 6 dpff
Internal Exon Starts
Internal Exon Ends
Last Exon Starts
Methylation Ratio
MethylationRatio
Distance [nt]
C
eIF4A3 KD 8 dpff
Y14 KD 8 dpff
Internal Exon Starts
Internal Exon Ends
Last Exon Starts
Methylation Ratio
Distance [nt]
Exon Length Classes
Short Exon + Short Intron
Long Exon + Short Intron
Short Exon + Long Intron
Long Exon + Long Intron

Figure S4. Effects of EJC depletion are detectable at 6 dpff and EJC-dependent m^{6}A patterns at 8 dpff correlate with intron length. (A) Generalized additive models (GAMs) of methylation frequency across splice junctions in *eIF4A3* (RNAi) and *y14* (RNAi) animals at 6 dpff reveal no considerable changes in m^{6}A deposition compared to wild type (Fig. 2C). GAMs were fitted for exon length classes defined by length quartiles in WT. 95% confidence intervals of the model fit are depicted as shaded area; methylation frequency was tested in bins of 10 nt. **(B)** GAMs of methylation frequency ratios between wild type and *eIF4A3* (RNAi) or *y14* (RNAi), respectively, at 6 dpff revealed a localized increase in methylation frequency at exon starts. GAMs were fitted for exon length classes defined by length quartiles in WT. 95% confidence intervals of the model fit are depicted as shaded area; methylation frequency was tested in bins of 10 nt. **(C)** GAMs of methylation frequency ratios between wild type and *eIF4A3* (RNAi) or *y14* (RNAi), respectively, at 8 dpff stratified by exon and flanking intron length (short intron: < 200 nt; long intron: > 250 nt; exons with a with a flanking intron in the 200 – 250 nt transition zone were excluded; short exons in this graph represent the two shorter exon length quartiles combined (as compared to Supplementary Figs. 4A,B). Long exons represent the two longer exon length quartiles combined (as compared to Supplementary Figs. 4A,B). GAMs were fitted for exon intron length classes and revealed changes in m^{6}A deposition upon knockdown of the EJC at exon starts. This was more pronounced in the long-intron classes. 90% confidence intervals of the model fit are depicted as shaded area; methylation frequency was tested in bins of 10 nt.

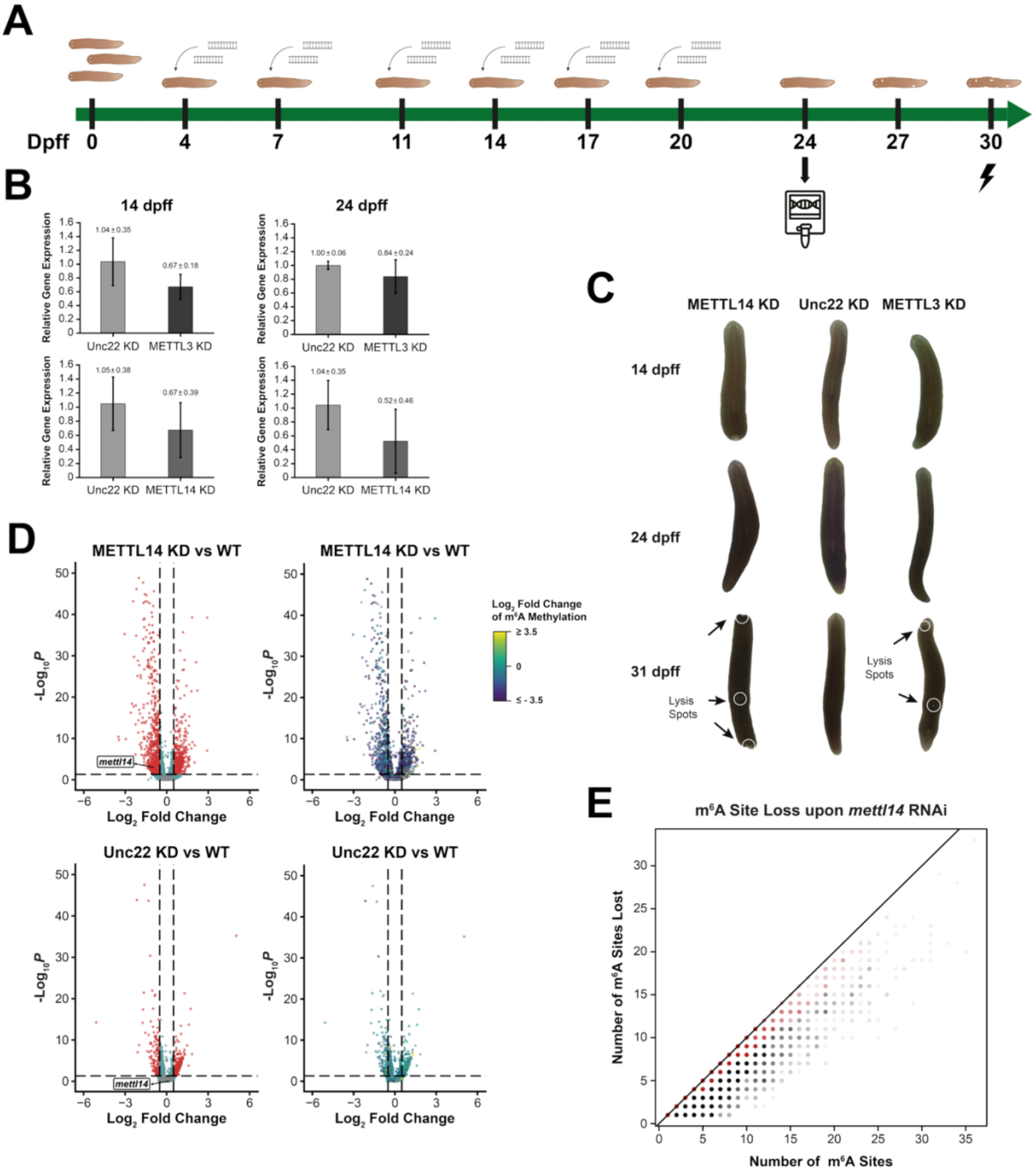
A
Dpff 0 4 7 11 14 17 20 24 27 30
B
14 dpff
24 dpff
Relative Gene Expression
1.04 ± 0.35
0.67 ± 0.18
1.00 ± 0.06
0.84 ± 0.24
1.05 ± 0.38
0.67 ± 0.39
1.04 ± 0.35
0.52 ± 0.46
Unc22 KD
METTL3 KD
METTL14 KD
C
METTL14 KD
Unc22 KD
METTL3 KD
14 dpff
24 dpff
31 dpff
Lysis Spots
D
METTL14 KD vs WT
Unc22 KD vs WT
-Log10P
Log2 Fold Change
mettl14
Log2 Fold Change of m6A Methylation
≥ 3.5
0
≤ -3.5
E
m6A Site Loss upon mettl14 RNAi
Number of m6A Sites Lost
Number of m6A Sites

Figure S5. Knockdown of MTC components via RNAi. (A) Experimental timeline for METTL14 knockdown followed by nanopore dRNA sequencing. Animals were fed dsRNA at the indicated time points (days post first feeding, dpff) leading to phenotypical changes starting from 27 dpff. For nanopore dRNA sequencing, RNA of control and knockdown animals was extracted at 24 dpff. **(B)** Initial qPCR validation of METTL3 and METTL14 knockdown efficiency. Relative METTL3 and METTL14 mRNA levels were analyzed at 14 and 24 dpff following RNAi treatment. Both knockdowns resulted in reduced target gene expression compared to the Unc22 control, with METTL14 showing a stronger reduction at 24 dpff. Bars show mean ± SE. **(C)** MTC knockdown causes progressive defects during planarian homeostasis. Representative images of animals subjected to RNAi against METTL14, METTL3, or Unc22 at 14, 24, and 31 dpff. While control animals maintained normal morphology, METTL3 and METTL14 depletion led to progressive tissue defects and the appearance of lysis spots (indicated by arrows and white circles) at later stages. **(D)** Volcano plots of gene abundance changes between *mettl14* (RNAi) and wildtype, as well as *unc22* (RNAi) and wildtype conditions. Genes with a significant ($p \leq 0.05$) $\log_2$-fold change ≤ -0.5 or ≥ 0.5 were considered as significantly deregulated (Left: red dots). On the right m^6A $\log_2$-fold change between respective conditions is displayed by dot color. **(E)** Scatterplot of the number of total vs lost m^6A sites, with m^6A sites being defined by a methylation rate ≥15% and loss of that site being defined as a reduction to 0%. We considered all transcripts that lost at least 80% of their m^6A sites (red dots) for analysis with the Bayesian Mixture model.

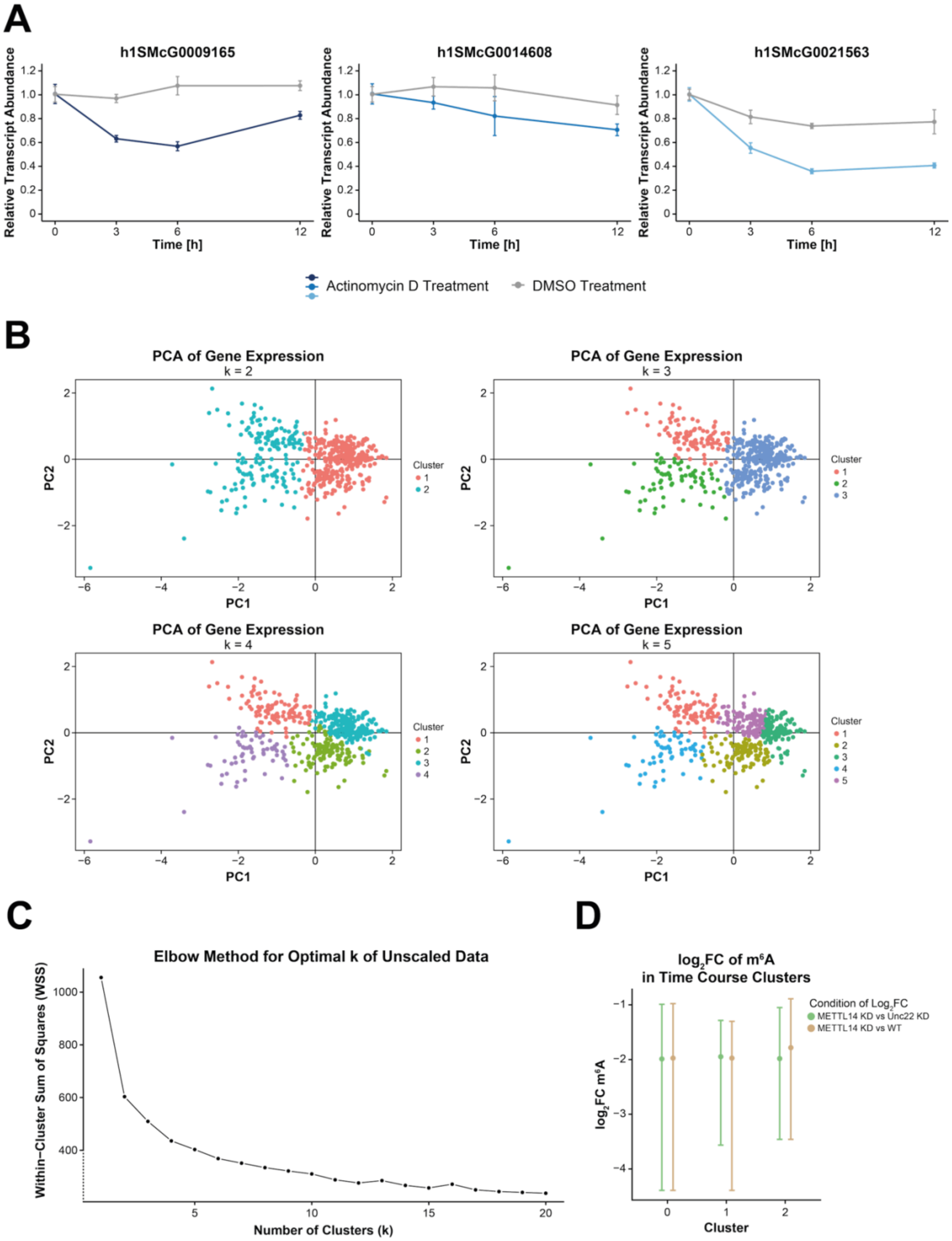
A
h1SMcG0009165
h1SMcG0014608
h1SMcG0021563
Relative Transcript Abundance
Time [h]
Actinomycin D Treatment
DMSO Treatment
B
PCA of Gene Expression
k = 2
k = 3
k = 4
k = 5
PC1
PC2
Cluster
C
Elbow Method for Optimal k of Unscaled Data
Within−Cluster Sum of Squares (WSS)
Number of Clusters (k)
D
log2FC of m6A
in Time Course Clusters
log2FC m6A
Condition of Log2FC
METTL14 KD vs Unc22 KD
METTL14 KD vs WT
Cluster

Figure S6. Validation of actinomycin D efficacy and determination of cluster numbers in time series analysis. (A) Wild-type worms were treated with actinomycin D (AD, blue) or DMSO (grey) and RNA extracted at 0, 3, 6, and 12 h. Intron-spanning primers targeting three transcripts (h1SMcG0009165, h1SMcG0014608, h1SMcG0021563) were used to quantify pre-mRNA by qPCR, with Ct values normalized to a spiked-in luciferase RNA control (3 pg, added prior to cDNA synthesis). Relative abundance is shown normalized to 0 h (mean ± SEM). **(B)** Principal component analysis plots for different cluster numbers displayed well defined clusters for k = 2, 3, and 4. **(C)** Within-cluster sum -of-squares (elbow-method) determined k = 2 as the optimal number of clusters for the time series analysis **(D)** Genes in time series clusters do not differ in loss of m^6A sites compared to genes not clustered in wildtype conditions. Dots represent the median; whiskers mark the first and third quartile. Significance was tested using Kruskal-Wallis test and p-values were corrected for multiple testing with the Bonferroni method.
